# Insight into lipid-based nanoplatform-mediated drug and gene delivery in neuro-oncology and their clinical prospects

**DOI:** 10.3389/fonc.2023.1168454

**Published:** 2023-07-06

**Authors:** Manasa Manjunath Hegde, Puja Sandbhor, Aishwarya J., Vikram Gota, Jayant S. Goda

**Affiliations:** ^1^ Manipal School of Life Sciences, Manipal Academy of Higher Education, Manipal, Karnataka, India; ^2^ Department of Biosciences and Bioengineering, Indian Institute of Technology, Mumbai, India; ^3^ Advance Centre for Treatment Research and Education in Cancer, Tata Memorial Centre and Homi Bhabha National Institute, Mumbai, India

**Keywords:** CNS tumors, immunotherapy, lipid-based nanoparticles, blood-brain barrier, clinical trail

## Abstract

Tumors of the Central nervous System (CNS) are a spectrum of neoplasms that range from benign lesions to highly malignant and aggressive lesions. Despite aggressive multimodal treatment approaches, the morbidity and mortality are high with dismal survival outcomes in these malignant tumors. Moreover, the non-specificity of conventional treatments substantiates the rationale for precise therapeutic strategies that selectively target infiltrating tumor cells within the brain, and minimize systemic and collateral damage. With the recent advancement of nanoplatforms for biomaterials applications, lipid-based nanoparticulate systems present an attractive and breakthrough impact on CNS tumor management. Lipid nanoparticles centered immunotherapeutic agents treating malignant CNS tumors could convene the clear need for precise treatment strategies. Immunotherapeutic agents can selectively induce specific immune responses by active or innate immune responses at the local site within the brain. In this review, we discuss the therapeutic applications of lipid-based nanoplatforms for CNS tumors with an emphasis on revolutionary approaches in brain targeting, imaging, and drug and gene delivery with immunotherapy. Lipid-based nanoparticle platforms represent one of the most promising colloidal carriers for chemotherapeutic, and immunotherapeutic drugs. Their current application in oncology especially in brain tumors has brought about a paradigm shift in cancer treatment by improving the antitumor activity of several agents that could be used to selectively target brain tumors. Subsequently, the lab-to-clinic transformation and challenges towards translational feasibility of lipid-based nanoplatforms for drug and gene/immunotherapy delivery in the context of CNS tumor management is addressed.

## 1 Introduction

### 1.1 World Health Organization classification of tumors of the CNS

Despite significant improvements in our understanding of the molecular basis of cancer, understanding the natural history of brain tumors is becoming a difficult task Intracranial neoplasms are a group of complex and heterogeneous lesions across the spectrum of benign and malignant tumors having varied treatments and clinical outcomes. The annual mortality rate attributing all malignant and non-malignant brain tumors and other CNS tumors account for about 23.79 per 100,000 as reported by the Central Brain Tumor Registry of the United States (CBTRUS) (1). The recent WHO classification of gliomas has incorporated immunohistochemistry in addition to tumor histology for characterizing gliomas. The present definition classifies diffusely infiltrative astrocytic tumors as being grade II (diffuse astrocytoma), grade III (anaplastic astrocytoma), or grade IV (tumors with microvascular proliferation and/or necrosis in addition to cytological atypia).These tumor have further been classified based on their IDH mutation status (2).

The identifying of regions with the distinctive histology for a particular tumor type is necessary for the categorization of central nervous system tumors since they frequently exhibit a wide range of morphological characteristics. Of all the brain tumors, glial tumors are the most commonly occurring and are categorized into astrocytoma, ependymomas, and oligodendrogliomas (3). Glioblastoma, also termed as Glioblastoma Multiforme (GBM), a Grade IV astrocytoma, constitutes 14% of intracranial tumor and 60% of astrocytic tumors. These tumors arise from glial cells within the central nervous system. Depending on the location of the brain tumor, the growth of tumors manifests as either localized or widespread symptoms, such as irregular headaches accompanied by aphasia and seizures (4). Oligodendrogliomas are another important adult tumor mainly originate from oligodendrocytes and are classified as grade II malignant tumor (5). Ependymomas are uncommon tumors of neuroectodermal origin that develop from ependymal cells in the spinal cord filum terminal, choroid plexus, or white matter of the brain (6). WHO has classified ependymoma grade- I tumors (myxopapillary ependymoma and sub-ependymoma) which are benign, slowly growing lesions. Grade III tumor is anaplastic ependymoma characterized by frequent mitosis, hypercellularity, and endothelial proliferation (7). Today, pediatric brain tumors are the leading cause of cancer-related death in children. One of the most common pediatric brain tumors is medulloblastoma, a primitive neuroectodermal tumor accounting for 20% of brain tumors in children (8). The biologically diverse group of medulloblastoma includes the following four molecular subtypes, namely WNT, Group 3, Group 4, and Sonic Hedgehog (9). The recent understanding of the molecular subgroups of medulloblastoma has resulted in shifting paradigms in not only the therapeutic strategy but also the prognosis of the disease.

### 1.2 Current challenges in CNS drug delivery

#### 1.2.1 Blood-brain barrier/blood-brain tumor barrier

The CNS and blood capillaries are connected by an extremely active and selective blood-brain barrier (BBB). It is the biggest barrier to getting drugs into the central nervous system through the blood circulation system. The BBB is a collection of highly specialized cells such as endothelial cells devoid of fenestrations, tight junctions, capillary basement membrane, astrocyte end-feet and pericytes that shields the brain from toxic substances in the blood and provides nutrients for function and helps in maintaining the brain’s homeostasis (10). Thus, even while the BBB is a natural defense mechanism, it also presents a considerable barrier to the systemic transport of many therapeutic medicines to the CNS. Similar to the blood-brain barrier, the blood-tumor barrier (BTB) is found between the microvessels and tumor cells in the brain. BBTB facilitates glioma cell migration to different regions of the brain and helps give nutrition and oxygen to the tumor. Utilizing this leaky BBTB linked to human brain tumors, anticancer drug delivery through nanocarriers may be accomplished by passively diffusing nanoparticles over free pharmaceuticals (11).

### 1.3 Strategies to improve the therapeutic efficacy in the brain

#### 1.3.1 Conventional therapies for brain tumor

Contemporary therapeutics in brain tumors include surgery, radiation, and chemotherapy, each treatment has its own benefits and drawbacks. Combination therapies are effective in maximizing both response and safety, and their regimen is carefully selected based on the patient’s age, health, and life expectancy as well as the rate of tumor progression, and stage of the tumor. Surgery is frequently combined with other therapies including chemotherapy and radiation for high-grade gliomas.

For brain tumors, there have been improvements in the delivery of local therapies. This includes the latest advancements in local therapeutic drug delivery, direct injection, convection-enhanced delivery (CED), and implanting drug-impregnated polymers. These techniques could enhance future therapeutic approaches, such as the implantation of microchips with drugs and local gene therapy (12, 13). Despite the drug being present in a biodegradable polymeric, interstitial diffusion of CW is constrained. The narrow volume of distribution inside the tumor and the peripheral brain tissue, as well as the high and diverse drug concentrations, have resulted in varied short-term therapeutic benefits and toxicity issues that prevent CW from being widely used in therapeutic settings (14).

## 2 Immune system and CNS tumors – landscape, interaction, therapy, and clinical challenges

### 2.1 Implication for CNS and immune system interaction

There is strong evidence to suggest that the “CNS is immune privilege” through the presence of immune cells (15–18). We summarized the key factors/determinants involved in the stimulation/inhibition of CNS immune responses during glioma pathogenesis (**Table 1**). These immune responses are produced *via* different mechanisms such as antigen in the brain, antigen presentation and APCs/microglia, T-cell trafficking, antibody penetration, and immunosuppression (immunosuppressive cytokines e.g. transforming growth-ß [TGF-ß], vascular growth factors [VGFs]) etc. Advance research on immune responses within the CNS have demonstrated that drainage of antigens in cervical lymph nodes through non-classical lymphatic pathways with cranial nerves cause induction of various immunological responses (cellular, humoral, innate) thus, resulting in trafficking of activated T-lymphocytes to the brain despite the presence of BBB (39–43). Moreover, antigens gain access through the convective flow of CSF and olfactory nerves across the cribriform plate into the systemic circulation and cervical lymph pathways. Subsequently, these antigens encounter B-lymphocytes and professional APCs in cervical lymph nodes and introduce them to circulating naïve T-cells, and lead to the activation of immune effector mechanisms. Thus, the activated lymphocytes *via* CNS get recruited at the site of inflammation although the native lymphocytes are unable to cross the BBB.

**Table 1:** Determinants of immunosuppressive tumor microenvironment in the gliomas.

Determinants	Immune response	Mechanism	Ref
TGF- ß EGF/EGFR/IL8,VEGF, COX2, FGF, IL6	Growth, invasion, and expansion of gliomas	1. Stimulating tumor-blood vessel formation and tumor metastasis by activating oncogenic genes e.g. Ras and MAPK, induction of hypoxia-inducible factor (HIF), PI3K/AKT and STAT-3 pathway *via* COX2 activation. 2. Reduce the intratumoral population of M1 phenotypes of microglia/macrophages	(19–23)
Transmembrane tyrosine kinase receptor c-Met ligand e.g. Monocyte chemotactic proteins (MCPs), Glial derived neurotrophic factor (GDNF) Chemokines and receptors (CCL5/CCR5, CCL2/CCR2, CXCR1/CXCR1)	Promote chemotaxis of immune cells Promote local immunosuppression	Act as chemokines for microglia and macrophages. Upregulation of CXCR4 through NF-κb pathway. Induce proliferation and migration of NKs, T-cells, microglia, macrophages, and DCs promoted *via* interaction with CCR1/2/3 receptors Recruitment of Tregs and MDSCs increased expression of matrix metalloproteases (MMP2, MMP9, MMP14)	(24–26) (27) (28, 29)
Glycoprotein-A repetition predominant (GARP), a surface molecule of regulatory tumor cells and T-cells), IL10 Indoleamine 2,3 dioxygenase (IDO-1), a tryptophan catabolism enzyme Arginase (produced by MDSCs) Programmed cell death protein and its ligand (PD-1/PD-L1) and Fibrinogen like protein-2 (FGL2)	Promote tumorigenesis and Immunosuppression Promote tumor progression through tumor immune escape Induce inflammation, and angiogenesis *via* promoting macrophages/microglia polarization Promote tumor immune escape	Induce differentiation of Treg, M2 phenotype generation (macrophages), suppression of effector T-cells Promote M2 phenotype generation of macrophages thereby inhibiting phagocytosis via STAT3 pathway activation, and induce expression of anti-inflammatory TGF-ß and reduce expression of pro-inflammatory molecules Treg, CD8T cells. Downregulate kynurenic acid metabolism through Treg-mediated Foxp3 apoptosis of CD4+T cells Reduce L-arginine concentration essential for normal T-cell functions *via* the production of nitric oxide (NO) using nitric oxide synthase (NOS) Induce expression of cytotoxic CD8-T lymphocytes and release anti-apoptotic signals inhibiting elimination of tumor cells, increase Treg, MDSCs expression, induce polarization of M2 phenotypes macrophages, and inhibit CD103+ DCs differentiation	(30) (31) (32) (33) (34, 35)
Glioma derived Macrophage colony-stimulating factor (M-CSF), Granulocyte-macrophage-colony-stimulating factor (GM-CSF), IL4, IL13 TGF- β	Promote glioma-associated macrophages (GAMs) polarization into M2 phenotypes	Promote differentiation of immunosuppressive M2 phenotypes of microglia and macrophages Activation of PI3K pathways and downstream transcription factor IRF4 Synergistically act with prostaglandin-E2 and inhibit MHC-I/II molecules expression on the glioma cells’ surface	(36) (37, 38)

During normal homeostasis inflammatory immune cells that cross the Virchow-Robin space are constrained in the perivascular space without surpassing the glia limitans (44). However, the integrity of the BBB is compromised during inflammation/diseased conditions, thereby permitting immune cell infiltration into the brain parenchyma (45). The complex interaction between resident immune cells like microglia, recruited macrophages, and lymphocytes from the periphery are key players in immunological responses in the CNS (46). *in-vitro* and *in-vivo* studies have shown the role of resident microglia on glioma progression *via* major histocompatibility complex class-I (MHC-I) and recruitment of CD8+ T-cells (47). Additional studies corroborating prior findings on the role of stimulated microglia as intrinsic immunotherapy candidates through efficient cross-priming of intratumorally injected exogenous antigen (OVA ovalbumin) to naïve CD8+ T-cells have been published (48).

### 2.2 Nano drug delivery bypassing the BBB

The CNS and blood capillaries are connected by the highly active and selective BBB. It acts as a barrier to drug delivery through the blood circulation system into the CNS. Most of the brain-targeting nanoparticles are given intravenously, which causes some of the drugs to reach other organs and cause toxicity (49). In contrast to systemic administration, local delivery provides several benefits, including avoiding the BBB and improving the therapeutic agent’s local bioavailability without producing systemic toxicity (50). A large spectrum of nano-platforms, including liposomes, polymeric NPs, micelles, protein nanocages, and inorganic NPs, are known for efficient drug delivery to the brain tumor. Nanoparticles enhance drug solubility, extend blood circulation half-life, and regulate drug release when used as drug delivery systems. Even though the BBB is intact, nanoparticles can be decorated with protein receptors and carriers that can mediate the transport of particular ligands and their contents. Additionally, the BBB membrane has a strong affinity for positively charged substances due to its negative charge, which may also cause cells to internalize substances. Therefore, these ligands might act as a conduit for NPs to cross the BBB (51, 52).

### 2.3 Different lipid-based nanoplatforms

Lipid-based or lipidic nanocarriers are widely used as drug delivery systems (DDS) since they have a variety of beneficial qualities, including high drug loading efficiency, low toxicity, biocompatibility, sustained release behavior, protection against drug degradation, stability, and suitability for drug delivery *via* various routes (53). Lipidic nanocarriers can be divided into several groups according to their physicochemical characteristics and technique of preparation: Liposomes are spherical vesicles with a lipid bilayer made of phospholipids. Niosomes are made of cholesterol and non-ionic surfactants. Transferosomes are similar to liposomes and are made of stabilized lipid matrix. Solid Lipid Nanoparticles have a solid lipid core. Nanostructure lipid carriers (NLCs) have a liquid lipid core encircled by a solid lipid core (54). Self-assembled spherical phospholipid bilayers known as liposomes have drawn a lot of interest as drug delivery vehicles for brain tumor therapy. Major benefits of liposomal drug delivery platforms include improved pharmacokinetic impact and selective accumulation in brain tumors by passive and active targeting. Clinical trials are being conducted on several liposome-based drug delivery systems. In 2011, the beginning of phase I/II clinical trial with 2B3-101 in patients with brain metastases from breast cancer or gliomas (NCT01818713). In this study, PEGylated Liposomal Doxorubicin formulation 2B3-101, coupled with glutathione and specialized transporters on BBB enhanced drug delivery to the brain.

Preclinical studies have shown that incorporating endogenous ligands or monoclonal antibodies onto the liposome surface is primarily attributed to the ability of these ligand to cross the BBB and subsequently bind to receptor overexpressed on GBM cells. Therefore, the improved targeting of GBM cells by these ligand-modified nanoparticles is due to their ability to permeate the BBB and reach the tumor site. A potential method for brain-targeted drug delivery is the active targeting of multifunctional liposomes. These liposomes can be used as targeted drug delivery systems for the treatment of brain tumors because of their simple and large-scale production capability, customizable structure, capacity to cross the BBB and preferred aggregation inside tumor tissue. In the subsequent part of the paper, we have discussed the applications of liposomes as a therapeutic moiety and a theragnostic in immunotherapy for CNS tumors.

### 2.4 Stimuli-responsive lipid nanoparticles

**Figure 1:**
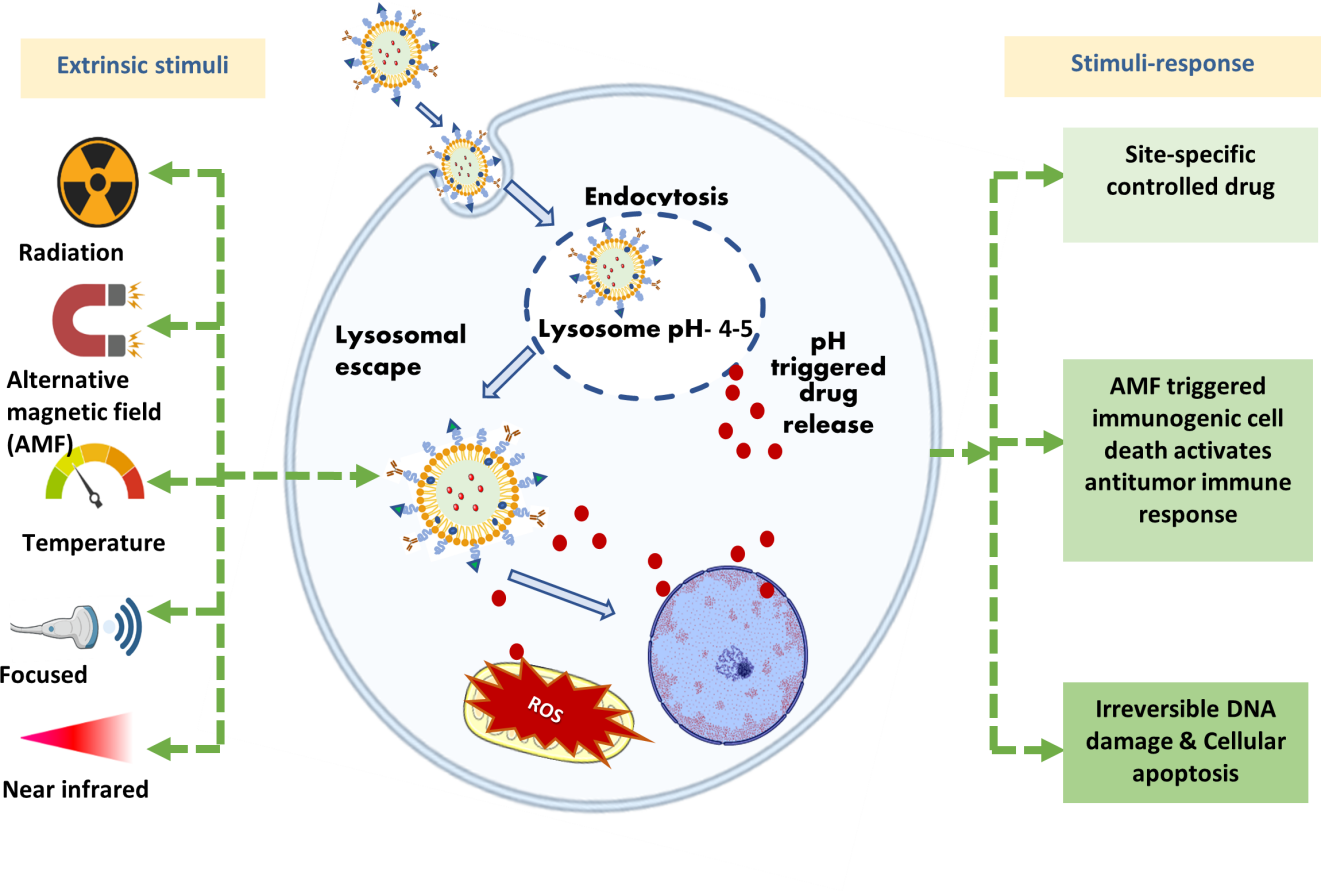
The stimuli-responsive multifunctional liposomes for drug delivery to CNS tumor cells for effective therapy by killing cancer cells by responding to external and internal stimuli.

Stimuli-responsive systems are showing promising results for site-specific delivery and release payloads. Many materials have been employed to make carrier systems, including lipids, polymers, and inorganic nanoparticles, all of which have been conferred stimuli-sensitive properties to achieve triggered release. The distinct characteristics of the tumor microenvironment may act as a site for an applied external stimulus (heat or light) or as an endogenous stimulus (pH, redox potential, or distinctive enzyme activity) to induce the regulated release of the drug as depicted in **Figure 1** (55).

### 2.5 Reassessing the immune microenvironment within the CNS tumors

**Figure 2:**
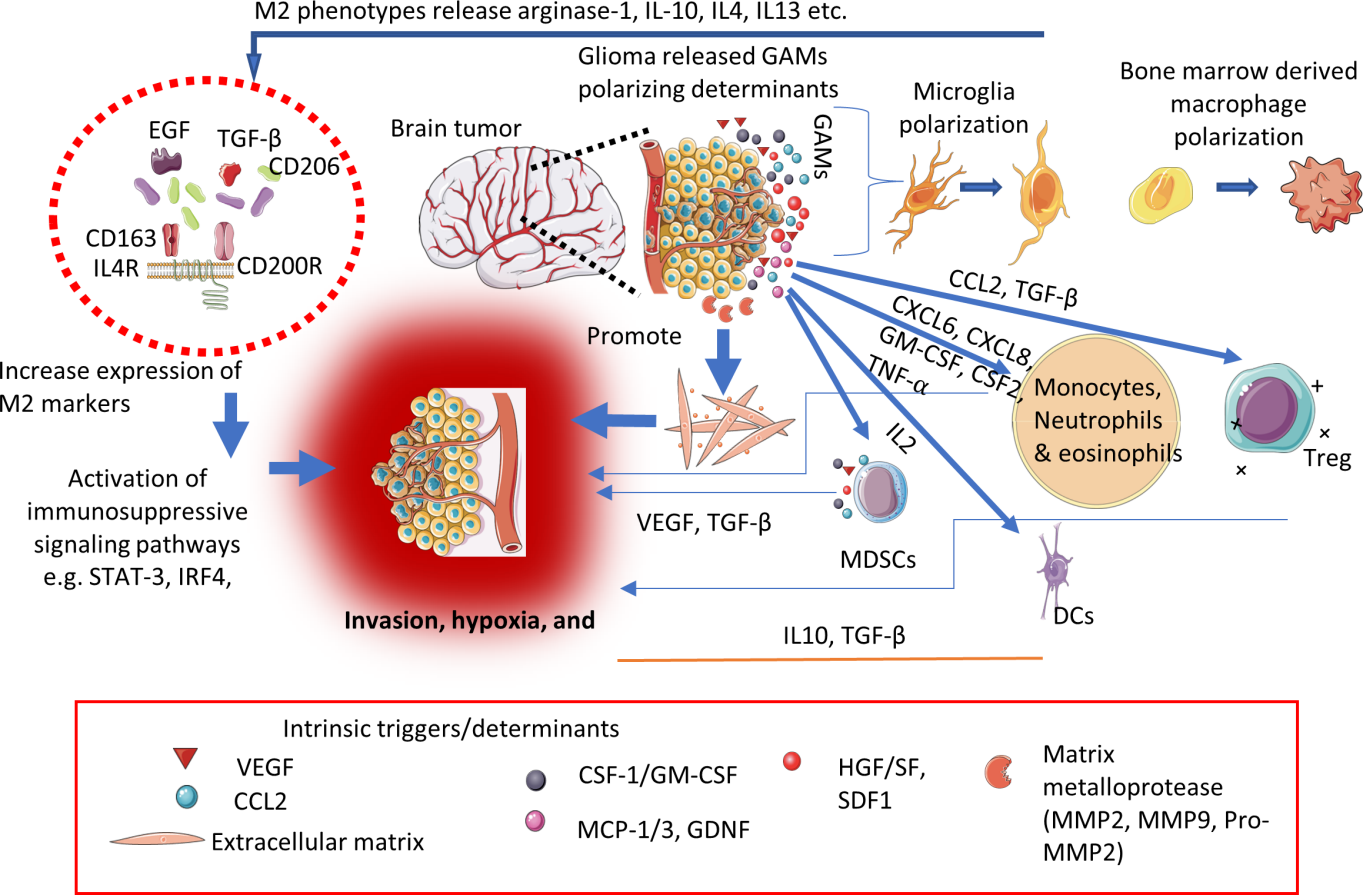
Schematic representation showing key determinants/triggers responsible for immunosuppressive microenvironment within the CNS tumors.

Glioblastomas are responsible for local immunosuppression through different immune escape mechanisms for instance, increase expression of co-stimulatory signals (CD80, CD40, CD86), and upregulation of MHC-II molecules etc. These molecules are essential for effective interaction, and communication between glioma cells and the host’s immune cells. Their interactions lead to preclusion of recognition and elimination of tumor cells by T-lymphocytes resulting in local immunosuppression (56–58). Furthermore, glioma tumor microenvironment (TME) abundantly constitutes immunosuppressive players such as TGF-ß, IFN-γ, cytotoxic T-lymphocytes [CTLs]) (59–61), regulatory T-cells (Treg), and dysfunctional NK cells (overexpressed ~12 times more in tumor cells than in the normal cells) etc. Moreover, the recruitment and prolongs survival of these immunosuppressive players supported *via* activation of high concentration of cytokines and tryptophan catabolic enzyme like indolamine 2,3-dioxygenase-1 (IDO1) thereby aid to sustained the immunosuppression (62–65). The evolutionary investigation in the mechanistic of glioma-associated immunosuppression aids in precise understanding of CNS tumor immunology and designing novel technologies targeting local immunosuppression. For instance, glioma-associated MØ/MG collectively called as GAMs, occupy about 15-30% of tumor mass depending upon the clinical stage of the gliomas (66). The extravasation and increased number of GAMs into the glioma eventually resulted in disruption of the BBB functions, and co-related with histologically aggressive tumors respectively (67). We represented the glioma-associated immunosuppressive microenvironment and its triggers/determinant in the **Figure 2**.

Further GAMs possess a significant degree of plasticity (M1 and M2 phenotypes) depending upon the type of stimulus (68–70). Various studies based on cell-surface markers and gene profiling at transcription levels are under investigation to gain a greater understanding of the function and potential of GAMs targeted immunotherapies against CNS tumors (71–73). Additionally, glycolysis, hypoxia, nutrient deficiency, and high concentration of lactate impair the effector immune cell functions in the TME contributing to local immunosuppression and subsequently contributing to tumor progression, and invasion. migration, angiogenesis, and resistance to conventional treatment modalities (74).

### 2.6 Immunotherapeutic strategies

**Figure 3:**
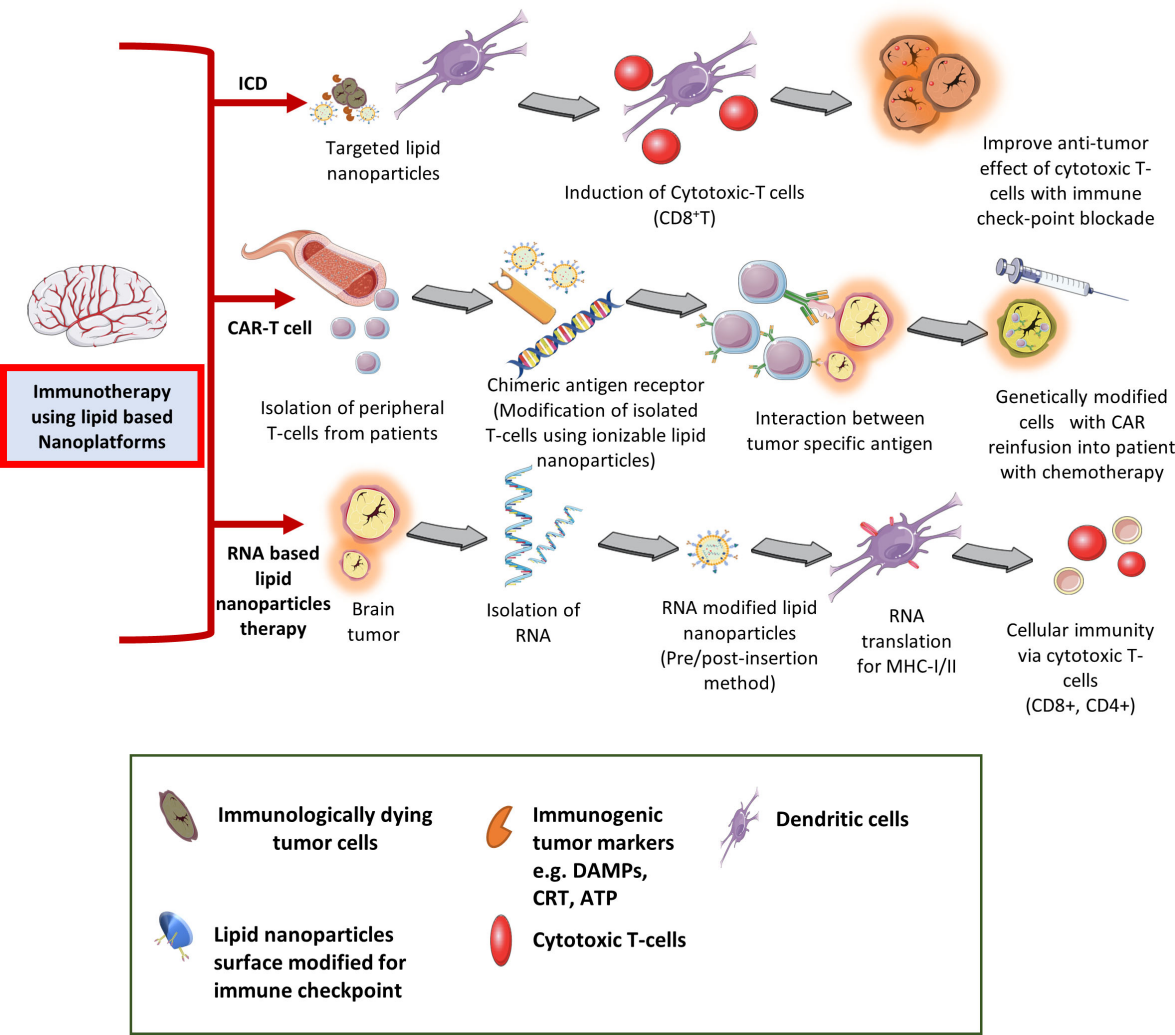
Different type of immunotherapy applications in treating CNS tumors using lipid-based nanocarriers.

Cancer immunotherapy is classified into active and passive immunotherapies (75, 76). Since past few decades, anti-cancer immunotherapies have experienced rapid clinical translation such as chimeric antigen receptor (CAR)-T cells therapy (use to enhance effective T-cells responses) targeting three major antigens i.e. human epidermal growth factor receptor-2 (HER2 or ERBB2), EGFRviii, and IL-12 receptor-α2 (IL-13Rα2) for brain tumors (77–79). However, as discussed earlier, the efficacy of cancer immunotherapy is limited by the factors by which cancer cells evade the host immune response *via* down-regulating surface antigen, and MHC-I expression by infiltrating inflammatory immune cells into the TME, tumor heterogeneity, and subsequently block the effector T-cells priming and activation (80). Current cancer immunotherapy based on activation/stimulation of key players of the immune system (e.g. cytokine therapy, adoptive T-cell transfer) and inhibition/elimination of immunosuppressive markers (e.g. checkpoint blockade [ICB] therapy). However, their clinical success found to be limited in the treatment of solid tumors, and brain cancers with immune response related adverse effects. To overcome these limitations existing immunotherapies with nanotechnology-based brain tumor targeting represent a captivating strategy (**Figure 3**). Recently, Chen and Cong in 2023 have provided a comprehensive review on the surface-modified nanoparticle platforms in anti-tumor immune response and immunotherapy (81). Furthermore, combination (e.g. chemotherapy, radiation) nano-theranostic and immunotherapy (e.g. macrophage polarization, dendritic cell targeting, NK cell therapies) have been more successful in clinical studies (82–84).

### 2.7 Current lipid-based nano-immunotherapies for the CNS tumors

Together with the development of smart nano-biomaterials and a series of promising clinical/preclinical studies (e.g. Pegylated liposomal doxorubicin in high-grade gliomas), a significant number of lipid-based nano-drug delivery systems have recently been appeared in anti-glioma clinical trials (85). In the following subsections, we described the recent development and translational barriers to lipid-based nanoplatforms in immunotherapy against CNS tumors.

#### 2.7.1 Immunogenic cell death by lipid-based nanoparticles

Various immunogenic factors during apoptosis of tumor cell have been identified to stimulate optimal antigen presentation to the effector T-cells such as damage-associated molecular patterns (DAMPs), calreticulin (CRT, a cell surface pre-apoptotic marker), ATP, and heat shock protein (HSP), and high mobility group box protein-1 (HMGB-1, a post-apoptotic protein), etc. (86). Several research groups have studied the pre-clinical potential of various lipid-based nanoplatforms (liposomes, nanodiscs, lipid-hybrid nanoparticles) for a combination of chemotherapy and immunotherapy against CNS tumors (87). For instance, Kadiyala et al. in 2019 demonstrated the immune-mediated anti-glioma potential of synthetic high-density lipoprotein mimicking nanodiscs (composed of 1,2-dipalmitoyl-sn-glycerol-3-phosphocholine [DPPC], 1,2-dimyristoyl-sn-glycerol-3-phosphocholine [DMPC] and egg sphingomyelin [SM]) loaded with docetaxel (DTX) and oligodeoxynucleotide (CpG a Toll-like receptor-9 agonist) as chemoimmunotherapy (DTX-sHDL-CpG) against glioblastoma. The combination of chemo-drug (DTX) with immunotherapy (CpG) exhibited an improved anti-glioma effect with no appreciable off-target toxicity. Authors further extended this application with adjuvant radiotherapy wherein the DTX-sHDL-CpG with radiotherapy significantly improved the survival benefits (~80% tumor regression) in GBM induced animals. Their findings confirm the role of immune system against tumor progression. Induction of potent cytotoxic CD8α^+^T cells lymphocytes (CTL) responses responsible for development of robust anti-tumor immunological memory against glioma recurrence (88). Consequently, in 2020 similar research group developed the sHDL nanodiscs containing a cocktail of immune checkpoint blocker anti-PD-L1 CpG, and cysteine-serine-serine (CSS) modified sequence (at N-terminus for conjugation to thiol-modified pyridyl disulfide modified phospholipids i.e. DOPE-PDP) neoantigen peptide as a personalized vaccination against syngeneic GL261 bearing mice model. They found that the nanodiscs platform in combination with a checkpoint blocker has the potential to induce neoantigen-specific CD8α^+^T cells infiltration into the glioma microenvironment. Collectively, their findings suggested the potential of lipid-based nanoplatform as personalized vaccination for immunotherapy against CNS tumors and other cancers (89). However, the clinical translation of the lipid-based nano platform for chemoimmunotherapy is still in its infancy because of many challenges such as scalability, safety and immunogenicity of the novel biomaterials, optimal ratio, synergistic index, dosing frequency/intervals of chemo-drug and immunotherapeutic to stimulate an immune response, etc. (29),. Thus, these factors need to be resolved for an efficient delivery of small molecules, drugs in CNS therapy using lipid nanoparticles.

#### 2.7.2 Lipid nanoparticle-based cancer vaccines in immunotherapy

Cancer vaccines are typically composed of antigens coupled with an adjuvant and induce *de novo* response against tumor-specific antigens (90). Local administration of vaccines leads to a cascade of innate inflammatory mediators *via* the release of DAMPs and chemokines gradient for chemotaxis of APCs at the site of inflammation (91, 92). As a result of the chemotaxis of APCs, antigens were pick-up before migrating to draining lymph nodes followed by their presentation and priming to an activated T-cell response against tumor-specific antigens (93). However, prophylactic and advanced stages of the cancers require multiple boosters over frequent intervals to years to confer adequate protection from immunosuppressive TME (94–96). Therefore, to target the tumor-specific antigens newer technologies need to be developed to harness the immune system in a personalized manner. Most prototype vaccines such as peptide, nucleic acids, mRNA, and DNA-based vaccines are associated with several limitations such as high immunogenicity, lack of stability, and unwanted degradation (97, 98). Alternatively, to prevent degradation and improve their stability before and after cell transfection various delivery vehicles have been developed (99). More recently, Mendez-Gomez and co-workers have developed a lipid nanoparticle multilayer mRNA backbone envelop (mRNA cancer vaccines) for gene transcript in pediatric high-grade glioma. They found localized uptake of RNA-NPs to myeloid cells in the KR158b glioma mouse model. Wherein the mRNA-modified lipid nanocarrier act by reprogramming the TME and inducing tumor-specific immunological response. Subsequently, this selective localization activates the DCs which supplement the regulatory myeloid cell population intratumorally to induce an antigen-recall memory with prolonged survival benefits. Additionally, this lipid nano-formulation received Food and Drug Administration-Investigational New Drug (FDA-IND) approval for a first clinical trial (IND number BB19304) in pediatric high-grade glioma patients (PNOC020 study NCT04573140) (100).

#### 2.7.3 RNA delivery using lipid-based nanoplatforms in immunotherapy

Lipid nanocarriers such as solid lipid nanoparticles and liposomes have been widely studied for the development of a sustained-release vehicle for therapeutic drug and gene delivery against gliomas (101). Various pre-clinical studies have provided a significant amount of toxicological data and offer a more straightforward path for the development of lipid nanocarriers for nucleic acid (e.g. RNA) delivery for further clinical translations (102). These RNA lipid nanoplatforms use the RES organs as more optimal locations for cancer vaccines to induce T-cells priming and APC transfection with antigen-presenting cells (e.g. liver Kupffer cells, splenic macrophages, dendritic cells) which phagocytize the RNA followed by activation of a T-cells against the desired epitope encoded by the mRNA (75). The RNA lipid nanocarriers were further modified with immunostimulatory cytokines encoded by targeted mRNA which are more promising and simple alternatives to dendritic cell-based cancer vaccines. Although lipid nanoplatforms for nucleic acid-based cancer vaccines mimic viremia through type-I interferon signature induction, they could not produce an anti-viral response against a viral antigen but redirect host immunity (innate and adaptive) against tumor mRNA antigenic molecules (103, 104).

Furthermore, the composition, size, and charge of lipid-based nanoparticles play an important role in eliciting immune responses. For instance, polar lipids are one of the major components of various lipid nanoplatforms and are significantly effective in mRNA transfection (105). The polar lipids composed of hydrophilic head groups attached to non-polar tails *via* linker bonds. The non-polar tail molecules from separate molecules joined to adjacent ones resulting in positively charged hydrophilic head groups that repelled each other and face along opposite sides. Thus, more liposomes join together and form a micelle-like structure having a positively charged outer surface followed by lipid layers either multilayers or single layers, and a positively charged inner core (106). Successively, RNA-loaded lipid nano-vehicles could also be formed by electrostatic interaction/complexation between positively charged lipid nanoparticles and negatively charged nucleic acid (e.g. RNA) (107, 108). An interesting proof-of-concept study of the intranasal DOTAP liposomes targeting the CFTR gene that overexpressed in lung parenchyma of cystic fibrosis (CF) patients demonstrated the safety in the first-in-human trial application (109). Subsequently, several research groups tried the DOTAP liposomes for the delivery of siRNA, and chemotherapeutic agents (paclitaxel and gemcitabine) for patients with advanced CNS tumors (110, 111). Sayour et al. in 2016 demonstrated the application of RNA-liposomal cancer vaccine encapsulated with personalized tumor-derived mRNA which represents tumor-specific transcriptome. This was achieved by isolation and extraction of total RNA (tRNA) from the brain tumor biopsied samples (n=500) to induce anti-tumor response against the murine adoptive cellular therapy for high-grade gliomas. The tRNA contains ribosomal and transfer RNA from which complementary DNA (cDNA) library was generated through RT-PCR on mRNA present from the initial tRNA. The cDNA was then transcribed *in-vitro* and amplified for the generation of multiple copies of mRNA mediate tumor-specific transfection of APCs. This result in antigen presentation onto the MHC-I/II (immunogenic tumor epitopes) *via* T-cell receptors (TCRs recognize most foreign and activated tumor epitopes and help the immune system to decide the best line-targets) thereby leading to activation of CD4 and CD8+ T-cells. Although, the study reported that tracking the antigen-specific immune response is more complicated when the immune reactive epitope is unknown (112).

Furthermore, Liu and co-workers in 2022 have developed the cholesterol and 1,2-dioleoyl-*sn*-glycerol-3-phosphoethanolamine (DOPE) lipid nanoparticles made up of cationic lipid with different ionizable amine headgroups (BAMPA-O16B, pKa 6.5) for endosomal escape of siRNA in GBM cell lines. Subsequently, they demonstrated that BAMPA-O16B/siRNA lipoplex is highly effective against the simultaneous blockade of two immune inhibitory markers involved in tumor-induced immunosuppression i.e. CD47 (a cancer biomarker overexpressed and prevents tumor cells phagocytosis through interaction with myeloid inhibitory receptor SIRPa) and PD-L1. This combination therapy leads to enhanced adaptive and innate anti-glioma immune responses in orthotopic glioma-bearing mice, due to synergistic activation of T-cell dependent downregulation of overexpressed target gene in the tumor (113). Grafals-Ruiz et al. in 2020 demonstrated the brain targeting peptides (apolipoprotein-E [ApoE] and rabies virus glycoprotein [RVG]) modified gold liposomes (30-50 nm) for siRNA delivery against dysregulated mRNA in GBM. Their study revealed the translational potential of miRNA based liposomal drug delivery for GBM and other CNS diseases (114).

Moreover, several lipid layers can be further modulated by layer-by-layer techniques to effectively traps the RNAs between lipid enveloped and prevent their degradation. However, precise control of the size of the RNA condensed lipid nanoparticles is necessary to avoid unintended biological activity such as transfection efficiency, cellular/intracellular uptake and retention, and toxicity profile (115). Thus, the lipid nano-platform for RNA delivery has great therapeutic potential however, stability and efficacy require more attention for clinical translation. Also, administration of lipid nanoparticles *via* the alternative route (e.g. intranasal, intrathecal, intraventricular) is highly desirable since these are effective strategies to circumvent the BBB, and can be explored further for future RNA-based nano-therapeutics against CNS tumors.

### 2.8 Specific cellular therapies using lipid-based nanoparticles

Cellular immunotherapy involves the administration of living cells (e.g. DCs) stimulating an anti-tumor response to the patients or adoptive transfer of cells (e.g. autologous or allogenic lymphocytes) having intrinsic anti-tumor potential (known as adoptive cell transfer [ACT]) to the patients. Although, the formidable barrier to these cellular therapies is presented by many solid malignancies *via* their immunosuppressive milieu resulting in impaired anti-tumor immunity (116, 117). Therefore, combination of different cellular therapies is under investigation to combat immunosuppressive TME (118, 119). However, high costs and a significant number of adverse events such as systemic autoimmune toxicity, and resistance to immunotherapy have been reported by many research groups (120–122). Subsequently, nanotechnology could help to solve this problem by using biocompatible, and inexpensive nano-carriers delivering rationally selected combinations of immunotherapeutic into the TME (123).

Recently, Shi and co-workers have demonstrated the T-cell targeting fusogenic liposomes coupled with 2,2,6,6-tetramethylpiperidine (TEMP) neutralizing reactive oxygen species. Fusogenic liposomes have potential in preventing T-cells from oxidative loss and allowing simultaneous MRI imaging. The T-cell’s functionality and proliferation depend on an optimal reducing microenvironment with redox balance between S-S and -SH on its surface membrane, however, the oxidative stress induced by tumor cells causes -S-S and -SH groups oxidation, and T-cells loss their functionality. Moreover, Fusogenic liposomes have a potential to improve the T-cells survival and proliferation, and may further provide opportunities for engineering of T-cells for cancer theranostic applications using lipid nanoarchitecture (124). Collectively, in the following sections we focus on these cellular lipid-based nano-immunotherapies in context of the CNS tumors.

#### 2.8.1 CAR-T cell therapy

In CAR-T therapy, a chimeric antigen receptor (CAR) is engineered T-cell with intracellular signaling domains for T-cells activation on antigen recognition when exposed to a particular tumor antigen of interest (125). Cancer-specific T-cells are produced *via* genetically modified T-cells using viral vector-based/non-viral nanoparticle-based systems. Widely used viral vectors (adenoviruses, retroviruses, and lentiviruses) for CNS tumors. Whereas non-viral vectors include various nano-platforms such as lipo/polyplexes, lipid/polymeric nanoparticles, etc. However, non-viral vectors have received greater attention due to their low immunogenicity, safety, efficacy, and ease of modification (126, 127). Recently Ferreras et al. reviewed the challenges and opportunities of CAR-T cell therapy in pediatric CNS tumors with special emphasis on circumventing these challenges through local and controlled delivery of tumor-specific effector immune cells using metal, polymeric, and lipid nanoplatform (128). However, very few studies have explored the role of lipid nanoparticles to enhance CAR-T cell therapy in brain tumors. In 2018, Zhang and their research group demonstrated the application of lipid nanoparticles (egg phosphatidylcholine, cholesterol, PEG (2000)-PE, and DSPE-PEG-maleimide) co-targeting (tumor-targeted peptide [iRGD] and PI3K inhibitor [CAL-101] to reduce the Treg cells and increased the number of effector T-cells) in reshaping the immunosuppressive tumor microenvironment for effective CAR-T cell therapy in a murine model of glioma. Their findings demonstrated that the preconditioning regimen used in the study with targeted nanoparticles transiently reshape the tumor immune microenvironment improving the success rate. Thus, this platform could be explored clinically to improve the CAR-T cell-based immunotherapy for the CNS tumors (129). Moreover, Billingsley and team have reported the orthogonal design of optimized lipid nanoparticle formulation *via* screening of sequential libraries of ionizable lipids for non-viral messenger (mRNA)-CAR-T cell therapy. The developed system has the advantage to improve mRNA delivery to T-cells with low cytotoxicity. Thus, suggesting the impact of formulation excipient optimization on lipid nanoparticles performance to improve the potency of treatment to deliver mRNA to primary human T-cells with comparable CAR expression by conventional electroporation (EP) method with low cytotoxicity (130).

Conclusively, there is an urgent need for increased translational strategies of these lipid-nanoplatform-based novel CAR-T cells therapy in the glioblastoma’s management. Several research groups have suggested that a judicious balance between the activation of CAR-T cells and cytokines secretions with acceptable toxicity, appropriate pre-clinical model recapitulating the host TME (syngeneic *vs* humanized mouse models), optimal characterizations (concentration and dosing frequency) of CAR-T cell therapy should be considered for accurate clinical translation to avoid tumor recurrence, toxicities, and resistance (131, 132). Subsequently, mathematical modelling and in-silico models could be useful tool to correlate the CAR-T cell therapy response and tumor regression. Additionally, the selection of T-cell subtypes having non-alloreactive phenotype like memory T-cells, broad therapeutic window of CAR-T cells therapy, and factors affecting it such as the density of tumor-specific antigens is necessary to be considered to enhance CAR-T cell efficacy (133, 134).

#### 2.8.2 Natural killer cell therapy

Natural killer (NK) cells are innate lymphoid cells located at the epithelial surfaces with selective cytotoxic potential leading to immunosurveillance upon exposure to pathogens or foreign substances (135). Furthermore, NK cells induce immunomodulatory functions by recruiting immune cells such as DCs, and T-cells and secreting cytokines (IFN-γ, TNF-α). However, NK cell therapy still faces considerable challenges in a clinical setting due to immunosuppressive TME that leads to decreased functionality, and poor infiltration and trafficking of NK cells into tumors (136). Various clinical studies have come up with positive outcomes from NK cells in combination with T-cells in pediatric brain tumors. The results from these clinical studies suggested that combinatorial therapies of NK cells with other cellular immunotherapies have the potential to enhance the effectiveness probably due to preventing tumor cells’ immune escape mechanisms (NCT02271711, NCT01804634, NCT02100891). Recently, Siegler et al. in 2017 demonstrated the CAR-T cells engineered NK92 cells as a combination therapy of paclitaxel (PTX) loaded cross-linked multilamellar liposomal vesicles (cMLVs). The liposomes composed of phospholipids i.e. 1,2-dioleoyl-sn-glycerol-3- phosphocholine (DOPC), 1,2-dioleoyl-sn-glycero-3-phospho-(10-rac-glycerol) (DOPG), and 1,2-dioleoyl-sn-glycerol-3-phosphoethanolamine-N-[4-(p-maleimidophenyl) butyramide (maleimide-head group lipid, MPB-PE) and have high specificity, selectivity, homing, and anti-tumor efficacy against human epidermal growth factor receptor-2 (HER2) and CD19 overexpressing solid tumor models (137).

Additionally, to track the NK-cells function as an anti-tumor candidate Yang et al. in 2020 reviewed the recent advances in non-invasive imaging methods like MRI, optical microscopy, and PET/SPECT wherein NK cells were tagged with fluorophores, radioisotopes/radiotracers, and paramagnetic nanoparticles against various cancer. The study addressed the major challenges to nanomaterial-based NK-cell therapy such as safety, and a detailed understanding of the mechanisms of NK cells in immunotherapy that needs further exploration (138). Moreover, due to the complexity of TME, the therapeutic potential of lipid nanoparticle-based NK cell therapy against CNS tumors is still under scrutiny to enhance the clinical effectiveness with acceptable off-target events. We anticipate that multifunctional lipid nanoparticles-NK cells technologies in combination with NK cell biology, and the complex interplay by TME could be targeted *via* promoting these engineered cells. Thus, the liposomal NK cells platform has a potential for treatment of other solid tumors including CNS tumors.

### 2.9 Cancer-initiating stem cells and gliomagenesis

Glioma stem cells (GSCs) or tumor-initiating cells (TICs) have been reported to be markedly resistant to conventional chemo/radiotherapy. They are responsible for tumor cells infiltration/proliferation, invasion, metastasis, and increase risk of tumor relapse, and mortality. The GSCs-mediated treatment resistance *via* upregulation of various stem cell markers (e.g. CD133), and ABCB1 transporters (MDR1) etc. were reported in brain tumors (139). Subsequently, high DNA repair ability of GSCs is driven through various mechanisms. For instance, stem cell markers integrin-α6 (receptor for the ECM protein laminin), and nestin (intermediate linker filament protein expresses by oligodendrocytes, astrocytes precursor and differentiated cells during embryogenesis). These stem cells markers were co-expressed with other proteins such a Proliferating Cell Nuclear Antigen (PCNA, an auxiliary protein use by DNA polymerase-δ during replication process), caspase-3, and Vascular Cell Adhesion Molecule-1 (VCAM-1, immunoglobulin mediate cell-cell interactions, and identified as a lineage-specific marker of neural differentiation, progenitor cells, and radial glia in the ventricular zone). They play an important role in maintaining connections between ECM matrix proteins in GSCs to support their propagation, adhesion, and invasion in perivascular niches and confer resistance to DNA alkylating agents and radiotherapy (140–142). In the following section we discussed about several GSCs targeted nanotechnology-based liposomal anti-glioma therapies for GBM treatment

#### 2.9.1 Liposomal nanoplatforms targeting GSCs

Various liposomal nanoparticles targeting GSCs either alone or in combination with external or internal triggers (e.g. hyperthermia, ultrasound, photodynamic/thermal therapy) have been extensively studied (143, 144). Furthermore, identification GSCs targeting specific stem cell-surface biomarkers (e.g. Aldehyde dehydrogenase [ALDHs], CD44, CD90, CD133), and unique signaling pathways (e.g. Wnt/b-catenin, Notch, TGF-β, Hedgehog) are under investigation to successfully eliminate GSCs to prevent tumor metastasis and recurrence (145). *In vitro* studies of CD44 targeted lipid nanoparticles modified with hyaluronic acid has been shown to improve therapeutic ratio against GBM. The hyaluronic acid acts as a biomimetic ligand to CD44, selectively targeting CD44 overexpressing glioma cells thereby improving anti-tumor efficacy of doxorubicin (146).

Studies have demonstrated GSCs transdifferentiating into endothelial cells and/or pericytes, and have been reported to promote vasculogenesis (the ability of tumor cells to form the embryonic circulatory system with new blood vessels using pre-existing vasculature) through upregulated VEGF (147, 148). Furthermore, involvement of multiple mechanisms/pathways were studied by many researchers in *in-vivo* and clinical samples using lineage-specific fluorescence reporter (149–151). For instance, recruitment of GSCs towards the SDF1-CXCR4 axis and subsequent activation by transforming growth factor-β (TGF-β, promoting cancer stemness and tumor metastasis), and hypoxia-inducible factor-1 (HIF-1α, a transcriptional regulator for hypoxia-induced gene expression important in maintaining cell cycle quiescence and promote angiogenesis etc. Their findings demonstrated the elimination of GSCs differentiated pericytes (having the same genetic alteration as glioma cells) helps in tumor regression. These studies advance our understanding of the plasticity of GSCs and may facilitate the designing of GSCs-targeted clinical nano-therapeutic strategies, and drug development to suppress glioma progression. In 2014 Li et al. reported the application of multifunctional liposomes co-loaded with paclitaxel and artemether in reducing the vasculogenic mimicry (VM) through induction of VM channels (composed of genotype-transformed tumor cells supplying nutrients to tumor cells and promoting their recurrence), and activation of apoptotic pathways for GSCs growth inhibition *via* downregulating expression of MMP-9, HIF-1α, and VEGF against invasive brain tumors (152). Moreover, studies have demonstrated that the overexpression and interaction of tumor biomarkers such as *integrin* α_v_β_3_ with ECM through tripeptide Arg-Gly-Asp (RGD) exhibited crucial effects on tumor cell proliferation, and invasion. Subsequently, nano-carriers targeting tumor vasculature have been developed that bind to the receptors overamplified during tumor angiogenesis like integrin, VEGF, and VACM-1 etc (153). Consequently, cRGD peptides have been widely investigated as a ligand for integrin using targeted anti-glioma strategies. For instance, Sofias et al. developed liposomes decorated with cRGD and oil-in-water nano-emulsion for ligand-mediated accumulation *via* immune cell phagocyte hitchhiking in GBM induced mouse models. The authors have demonstrated the real-time nanoparticle targeting kinetics, and their specificity to circulatory, and tumor-homing immune cells (e.g. monocytes, macrophages, neutrophils, and lymphocytes) using PET/CT imaging integrated with flow cytometry and intravital microscopy (154).

However, a better understanding of GSCs and their properties could support the development of personalized anti-tumor nanodrug therapies to fulfil unmet clinical needs. For instance, Wang et al. in 2009 has demonstrated the siRNA-modified nano drug delivery platform downregulating expression of HIF1*α in animal studies.* Their findings revealed the potential of anti-HIF1*α* siRNA-based integrin targeted multifunctional nanocarrier knockdown of HIF-1*α* expression in human glioma xenograft mouse models. Furthermore, Sakurai et al. in 2014 reported the pH-sensitive cationic (YSK05) liposomal siRNA nanoparticle (MEND) modified with cRGD for tumor endothelial cells (TECs) gene silencing, and for VEGF as an anti-angiogenic therapy (155). Recently, dual ligands targeted delivery strategies have shown great potential in the delivery of therapeutic agents to tumor cells for CNS tumor treatment as a means of circumventing the BBB compared to a single ligand modification system (129, 156). Recently a combination of integrin α_v_β_3_ and lactoferrin receptors (overexpressed in glioma cells and cerebral microvascular endothelial cells) targeted RGD-modified docetaxel (DTX) pegylated liposomes (RGD-Lf-LP-DTX, 140 nm size) for treatment of glioma have also been developed (157). These dual ligand modification plays an important role in increasing selective retention, and accumulation of the drug in orthotopic brain tumor model. The liposomes had shown anti-glioma effect with significantly improved survival of mice (32 days) compared to control (20 days), single ligand modified liposomes (28days), and unmodified drug-loaded liposomes (21.5 days) (157). Although, novel lipid nanoplatforms are reported to be effective against CNS tumors, interaction between nanoparticles and biological fluids can modulate the pharmacokinetics, brain uptake, and organ distribution thereby dampening of therapeutic effects (158, 159). Therefore, preclinical studies in orthotopic or patient-derived xenograft models (PDX) with robust clinical findings should be undertaken for translation of novel lipid nanoplatform systems against the CNS tumors (160).

### 2.10 Limitations and challenges in clinical translation of lipid-based nanoparticle drug delivery systems in CNS tumors

The primary treatment method for CNS tumors continues to be conventional surgery together with radiotherapy and concurrent chemotherapy, however, it offers little promise and runs the risk of tumor recurrence. Finding NP-based drug delivery systems that deliver medications directly to the cancer location is urgently needed, given the other numerous drawbacks of this therapeutic strategy. Recent advances in the molecular characterization of brain tumors have led to the development of targeted therapies. Despite the novel therapeutic intervention, the bottleneck arises in the clinical translation of drugs and their derivatives. The most common problems faced during drug delivery are; The inability of the drug to cross the BBB; poor specificity of the drug; short half-life or circulation time and physical and/or chemical interaction of the drug with the circulating proteins and brain tissue components etc (161–164). Therefore, newer and improved drug delivery methods need to be developed for the targeted delivery of the drugs to tumor cells while sparing healthy tissue.

#### 2.10.1 Safety considerations of lipid-based drug delivery and overcoming challenges in a clinical setting

**Figure 4:**
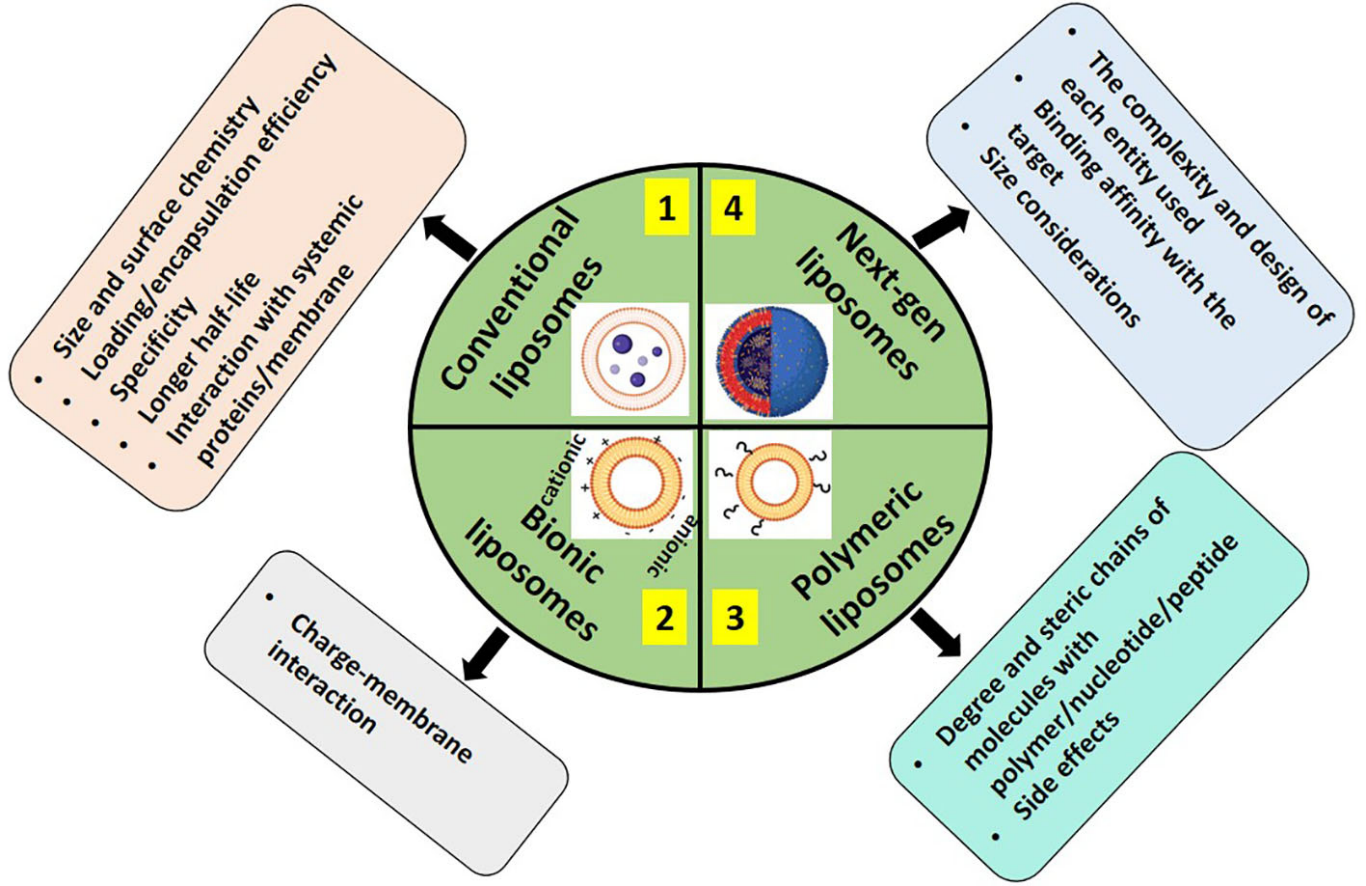
Considerations in using different types of liposomes 1. Conventional (encapsulated with a chemotherapeutic agent (165, 167–169), 2. Bionic; either cationic or anionic with the drug of interest (108, 170–172), 3. Macromolecule formulated; for example, PEGylated (172), and 4. Next-gen containing targeted ligands towards receptors that are typically overexpressed in case of brain cancers for better clinical utility (166, 173, 174).

Lipid-based drug delivery systems are promising candidates for addressing these needs for treating brain tumors. Encapsulating drugs in liposomes would be beneficial as they can cross the blood-brain barrier (BBB) more efficiently. However, liposomes can be difficult and expensive to manufacture on a large scale. Therefore, there is a need for alternative lipid-based drug delivery systems that can encapsulate drugs just as well but at a lower cost. Modifications of conventional chemotherapeutics or immune therapeutics have made drug delivery feasible and targetable in recent years in treating C high-grade gliomas (165). The pharmacokinetics of conventional liposomes after intravenous injection are very poor and are characterized by rapid systemic clearanced by plasma proteins and phagocytosis and removal by the liver, spleen, and other reticuloendothelial organs (166) **Figure 4** depicts some of the considerations in lipid-based drug delivery systems to the brain.

#### 2.10.2 Clinical trials of liposome-based nanomaterials in CNS tumors

The use of liposomes has been linked with lower mortality rates and better patient outcomes in patients with brain tumors, eventually may become a standard mode of anti glioma therapy. Trials comparing chemotherapy and liposome-encapsulated doxorubicin have shown improved response rates and longer progression-free survival time for patients with glioblastoma multiforme (GBM). Another study showed that when delivered by liposomes, the chemotherapeutic agent tamoxifen could cross the blood-brain barrier more effectively than it could on its own. Koukourakis et al. investigated the clinical applications of radionuclide-labeled liposomal doxorubicin in both primary and metastatic brain tumors. radionuclide-labeled liposomal doxorubicin concentrated 13-19 times higher than the normal brain parenchyma. at the targeted tumor site, A Phase I and II trial by Hau et al. evaluated the role of PEGylated doxorubicin in high-grade gliomas and demonstrated moderate efficacy against recurrent gliomas, especially Grade III gliomas. In addition, PEG-DOX proved to be a safe treatment regimen with no significant side effects. The 6-month progression-free survival rate in the PEG-DOX arm was 32% compared to 20% in the standard temozolamide arm. Notably, approximately 5% of patients with Grade IV disease and about 40% of patients with Grade III disease were stable for up to 160 weeks after the initiation of therapy and exhibited long-term survival (175). In addition to better clinical response, a few common adverse effects like hand foot syndrome and stomatitis were observed due to increased circulation time of the formulation.

PEG-Dox liposome (2B3-101) have been clinically evaluated in phase I and II trials in high-grade gliomas (HGG’s). Among 24 HGG patients, 54% had stable disease, and a 3-month progression-free rate of 33% (176). Based on the first-in-human study 2B3-201 was considered clinically safe, with no serious adverse events reported, indicating the marketing potential of this product in the near future. Liposomal irinotecan (nal-IRI) have been evaluated in patients with recurrent high-grade gliomas., Patient treatment was stratified based on the UGT1A1 status. Homozygous WT patients were started at 120 mg/m^2^ IV with a dose escalation of 60 mg/m^2^ increments every 3 weeks and in heterozygous patients (HT) dosing began at 60 mg/m^2^, with dose escalation of 30 mg/m^2^increments. The MTD achieved was 120mg/m2 and 150 mg/m2 in WT and HT, respectively, with no major side effects reported (177). In another phase I and early efficacy trial, the same formulation was tested among patients with diffuse intrinsic pontine glioma (DIPG) with CED (Convection Enhanced Delivery). NaI-IRI was administered directly into the tumor using CED-post-radiotherapy (178). Similarly in a trial, when NaI-IRI was administered intravenously was considered safe with no anticipated toxicity among GBM patients. Notably, UGT1A1 genotype did not influence PK profile or correlate with toxicity (177).

In 2022, Kasenda and group, for the first time in a phase I trial, clinically assessed the capacity of EGFR-targeted immunoliposomes in delivering the cargo to brain tissue in patients with relapsed EGFR amplified glioblastomas. C225-ILs-dox-PEGylated liposomal doxorubicin containing the Fab fragment of anti-EGFR antibody CC25(Cetuximab), was administered at a dose of 50 mg/m2 intravenously, on day 1 of each cycle, every 28 days Anti-EGFR ILs-dox were infused intravenously with dose escalations of (doxorubicin 5 mg/m2, 10 mg/m2, 20 mg/m2, 30 mg/m2, 40 mg/m2, 50 mg/m2, and 60 mg/m2) once every 4 weeks for a maximum of six cycles in 26 patients. The MTD achieved was50mg/m2.Although no significant side effects were observed, one patient developed severe pneumonitis (179). **Table 2** summarizes the ongoing and completed clinical trials utilizing lipid based drug delivery system.

**Table 2:** Clinical trials – past, present, and future of lipid-based drug delivery systems in CNS tumors.

Disease condition	Type of liposome and treatment strategy	NCT Number	Trial Phase	Trial status
Recurrent High-Grade Glioma	Nanoliposomal CPT-11 (starting dose 120 mg/m^2 (wild type) or 60 mg/m^2 IV q3 weeks.)	NCT00734682	I	Completed (2008-2014)
Glioblastoma	Radiotherapy (60Gy/30 fractions plus 75mg/m^2^ TMZ daily. Pegylated doxorubicin (once prior to radiotherapy and on day 1 and 15 of each 28-day cycle 4 weeks post radiotherapy)	NCT00944801	I-II	Completed (2009-2014)
Primary brain tumors	Marqibo (Vincristine Sulphate liposome) – IV route 28-day cycle	NCT01222780	I	Completed (2010-2014)
Refractory primary malignant gliomas (II and IV)	2B3-101(Glutathione Pegylated liposomal doxorubicin hydrochloride) 2B3-101(40 mg/m2 every 3 weeks.)	NCT01386580	I and II	Completed (2011-2014)
Recurrent High-grade glioma	Nanoliposomal irinotecan (MM-398 or ONIVYDE) 3 + 3 dose escalation model from 20mg to 680mg with tumor size variation)	NCT02022644	I	Active, not recruiting (2014-
Recurrent Glioma (II and IV)	Rhenium-186 NanoLiposome (dose escalation from 1mCi to 41.5mCi) in Phase I and 22.3 mCi in Phase II	NCT01906385	I and II	Recruiting (2015-
High grade glioma	Carboxylesterase-expressing Allogeneic Neural Stem Cells with liposomal irinotecan	NCT02192359	I	Active, not recruiting (2016-
Diffuse Intrinsic Pontine Glioma (DIPG)	Nanoliposomal irinotecan (nal-IRI)	NCT03086616	I	Completed (2017-2021)
High grade glioma	Doxorubicin-loaded Anti-EGFR-immunoliposomes (C225-ILs-dox), C225-ILs-dox administered at a dose of 50 mg/m2. i.v., on day 1 of each cycle, cycle length was 28 days, in a total of 4 cycles	NCT03603379	I	Completed (2018-2020)
Paediatric high-grade gliomas and Adult GBM	RNA-Lipid particles (Loaded with autologous total tumor mRNA and pp65 full length (fl) lysosomal associated membrane protein (LAMP) mRNA)	NCT04573140	I	Recruiting (2021-
Recurrent high-grade glioma (EGFR mutant)	Visudyne (liposomal verteporfin)	NCT04590664	I and II	Recruiting (2021-

(Data meta-analyzed using clinicaltrials.gov.in with the search keywords- neural/glial/CNS tumors/lipid/liposomes therapy)

### 2.11 Addressing the bottleneck in clinical translation of liposome drug delivery system in CNS tumor management

Despite providing a multitude of applications in biology apart from cancer treatment, the delay in clinical translation of liposomes from preclinical to clinical is contributed by many issues such as pharmaceutical development being limited by cost and cumbersome quality assurance protocol (180). The complexity of multifunctional liposomal formulations complicates the larger scale manufacturing, cost, etc, in an industrial setting and Pk/PD, toxicity, and biosafety evaluation in research settings (181, 182). Clinical trials are more complex to account for groups for each entity of multifunctional nanoformulation, the discrepancy in the translation of therapeutic efficacy *In vivo* to humans (183, 184). Patenting and Copyrights (part of Intellectual property rights) contribute to the cost of development depending upon the complexity of formulations, design, composition, etc which may overshadow the commercial attractiveness, and the multiple patents over a given formulation might call for a need for cross-licensing arrangements (185–188).

For nano-liposomes to translate from a research lab to a clinical setting, challenges still need to be overcome. For instance, the safety and toxicity of liposomal formulations need further optimization, as the chemical method of synthesizing lipid-based NPs utilizes toxic organic solvents which are difficult to remove otherwise; secondly, long-term *In vitro* and *In vivo* toxicity and safety evaluation is required in case of nanomaterials due to their slow rate of metabolism, and; thirdly, the clinical translation of polymers, such as PEG mainly addresses such problems as their interaction with cell membranes results in low drug retention, increased toxicity, and rapid clearance from blood circulation. First, it is necessary to decrease the cytotoxic immune response and lengthen blood circulation by altering the surface of cell membranes, such as cationic polymers, peptides, etc. To increase the stability and effective crossing of NPs *via* the blood-brain barrier *In vivo*, it is also vital to optimize the size of nanoplatforms. Although new liposomal-based drug delivery systems have been well explored and established in preclinical animal models, these liposomal pharmaceutical products may not provide promising therapeutic effects in clinical trials. Next-generation development of liposomal-based drugs, the comparison of drug circulation time in blood, drug accumulation in tissues, and possible toxicity between conventional vesicles and new classes of liposomes should be investigated in preclinical animal models for better clinical utility.

## 3 Conclusion

**Figure 5:**
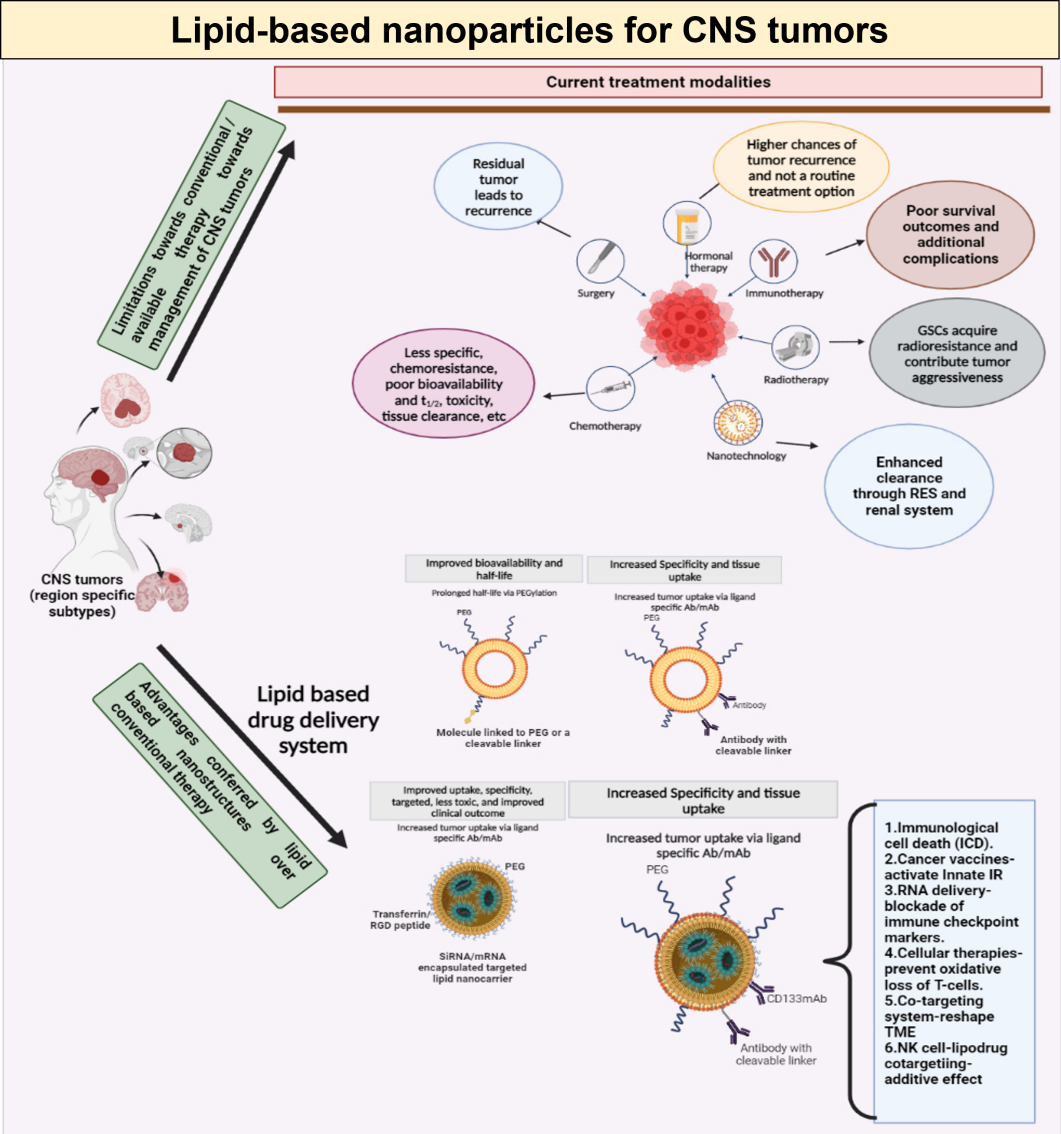
Schematic representation of limitations in conventional therapeutic management of CNS tumors, and the benefits offered by lipid-based drug delivery systems, with special emphasis on immunoliposomes.

Due to their heterogeneity, infiltrating nature, and inherent resistance to radiation and chemotherapy, gliomas, are considered the least treatable cancer. Hence, using targeted drug delivery systems that increase drug concentration in tumor tissue while avoiding systemic adverse effects is necessary for developing smart and effective therapeutics. One of the most promising approaches in treating brain tumors is nanomedicine-based delivery of drugs or biologics (immunotherapy). The advantages offered by lipid-based nanotherapeutics, especially the immunoliposomes is depicted in **Figure 5**. Nevertheless, many hurdles, such as the highly repressed immune microenvironment, the restricted drug transport to the central nervous system, and others have hampered the huge promise of chemotherapy and immunotherapy in these tumors. With the evolution in nanotechnology, drug delivery platforms have been created which can transport not only chemotherapeutics but also biologics like immuno therapeutic agents that work synergistically at the targeted site. The distribution of therapeutic compounds to brain tumor sites can be aided by the development of colloidal nanocarriers such as liposomes that release their payload inside the tumor microenvironment after selectively targeting immune cells.

## Author contributions

MH and PS share equal authorship. MH, PS and JA contributed to conceptualization, resource collection, and writing the original manuscript. VG contributed to reviewing, editing and supervising. JG did conceptualization, visualization, supervision, reviewing and editing the manuscript. All authors contributed to the article and approved the submitted version.
